# Supplementary figures and images for: TM2D genes regulate Notch signaling and neuronal function in Drosophila
Source: PLoS Genet. 2021 Dec 14;17(12):e1009962. doi: 10.1371/journal.pgen.1009962 (PMC8714088; doi:10.1371/journal.pgen.1009962)

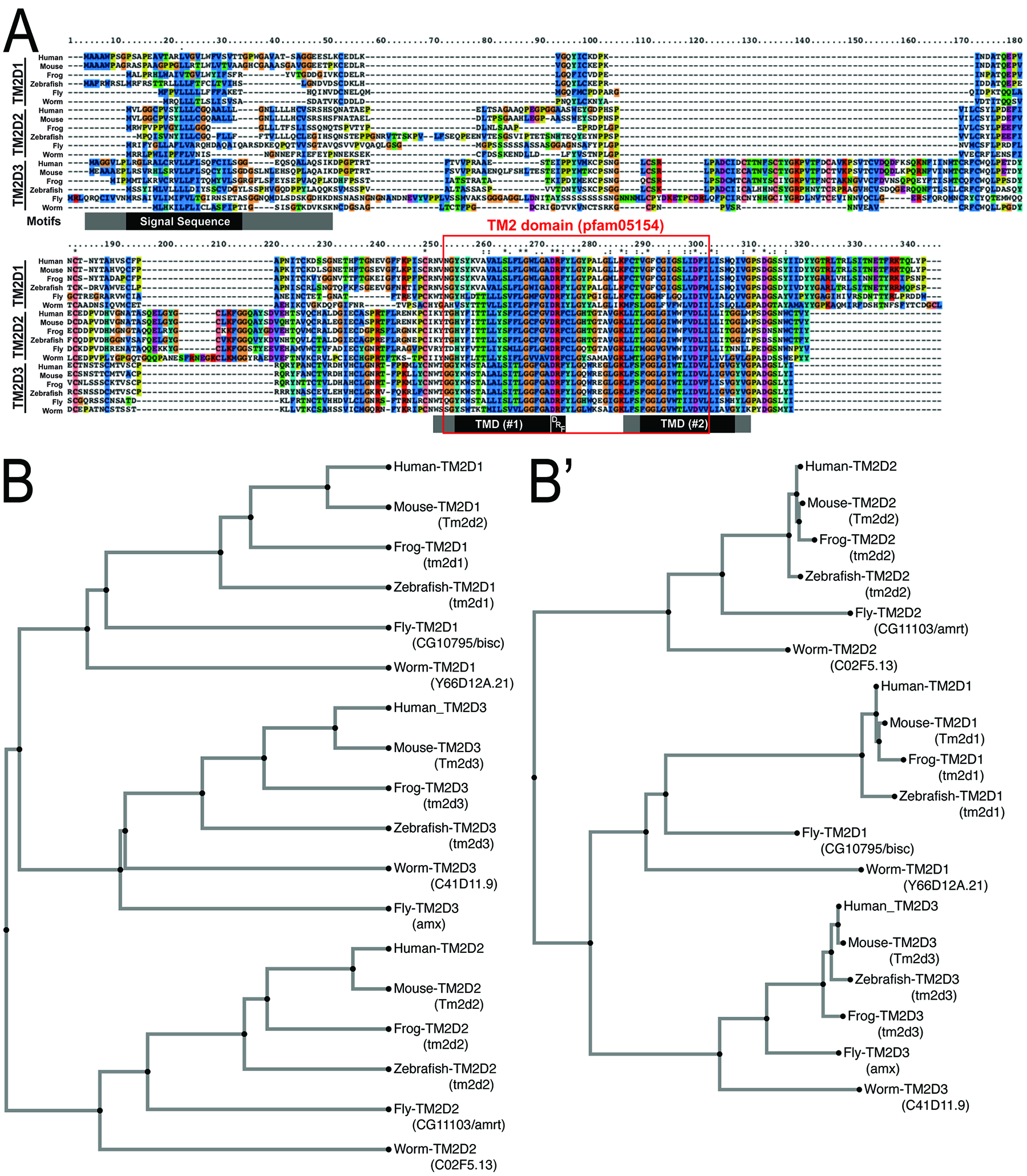

Supplement: S1 Fig — (A) Protein alignment of Human TM2D proteins across multiple species (human, mouse, frog, zebrafish, fly, worm). TM2 domain (red box, pfam05154) is highly conserved among the proteins and across species. C’ terminus of the proteins are also well conserved across species. Black bars denote the regions that are commonly annotated as Signal Sequence, TMD (Transmembrane domain, #1), DRF or TMD (#2) in all human TM2D1-3 proteins (consensus regions) based on Uniprot (https://www.uniprot.org/). Gray bars show regions that have been annotated as Signal Sequence, TMD (#1) or TMD (#2) in one or two human TM2D1-3 proteins. (B-B’) Phylogenetic analysis of TM2D family genes. (B) shows the phylogenetic tree based on UPGMA (unweighted pair group method with arithmetic mean) method, and (B’) shows the tree based on the Neighbor-Joining method. In both cases, orthologs across different species are shown to be more closely related to each other than the three genes in each species. (TIFF) [file pgen.1009962.s001.tiff]

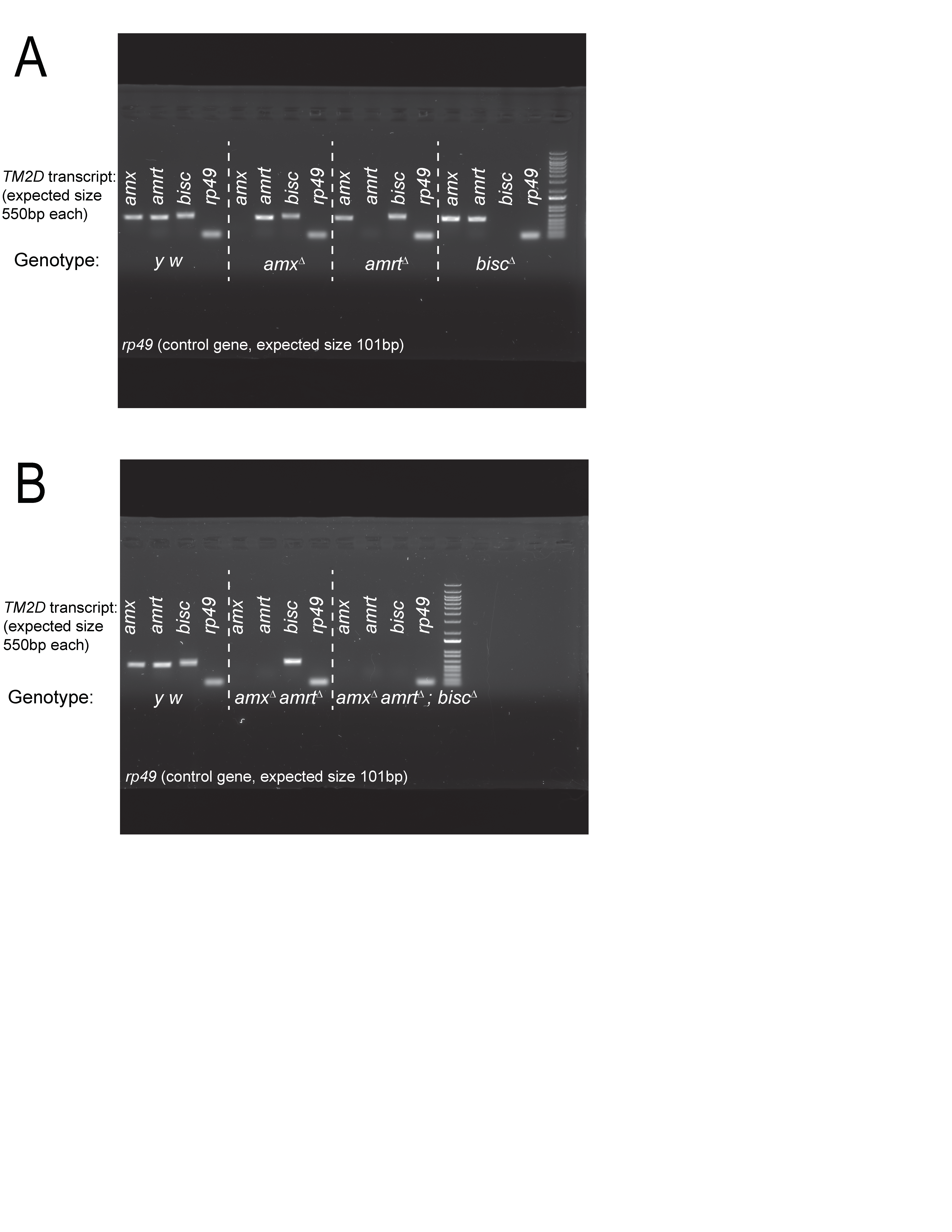

Supplement: S2 Fig — Reverse transcription followed by PCR (RT-PCR) to verify loss of TM2D gene transcripts in mutant fly lines. mRNA was isolated from animals homozygous for their respective alleles. (A) Single mutant lines lack their appropriate gene transcript while other TM2D transcripts are unaffected. (B) amx amrt double mutants express bisc. TM2D triple mutants lack all transcripts. rp49 is a house-keeping gene used as a control for the reverse transcription reaction. (TIFF) [file pgen.1009962.s002.tiff]

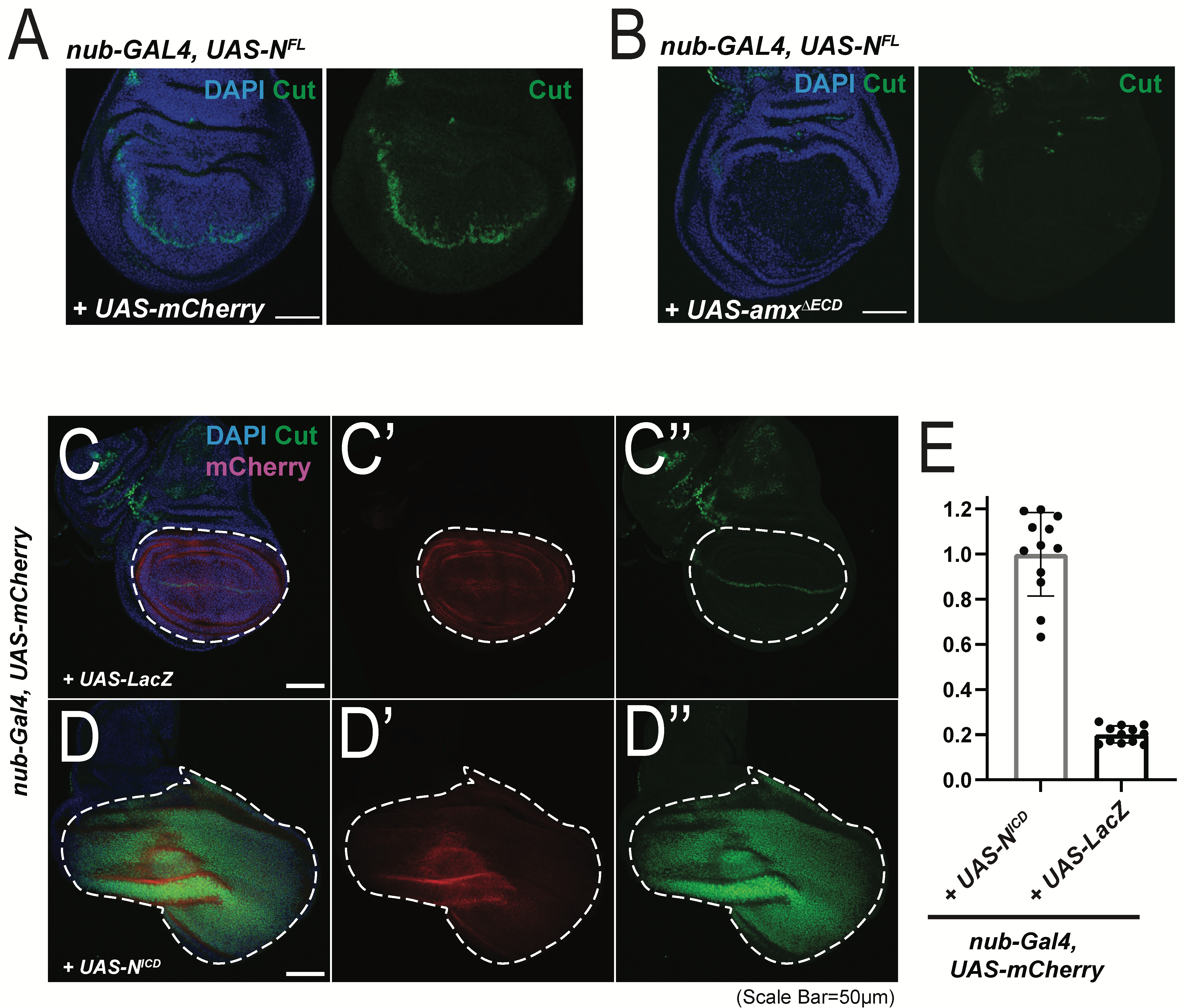

Supplement: S3 Fig — (A) Overexpression of full-length Notch in the developing wing pouch via nub-GAL4 causes a minor upregulation of Cut expression close to the wing margin, likely reflecting the availability of ligands within the wing pouch. (B) AmxΔECD inhibits the increase of Cut expression induced by Notch as well as abolishing the normal expression levels of Cut, showing it is epistatic to full-length Notch. (C-D) Overexpression of NICD causes increased expression of Cut (green) (D-D”), compared to a control disc overexpressing LacZ, a neutral protein. (E) Cut expression is about 5-fold higher in NICD expressing wing discs compared to control discs expressing LacZ. Scale bars = 50 μm in (A-D). (TIFF) [file pgen.1009962.s003.tiff]

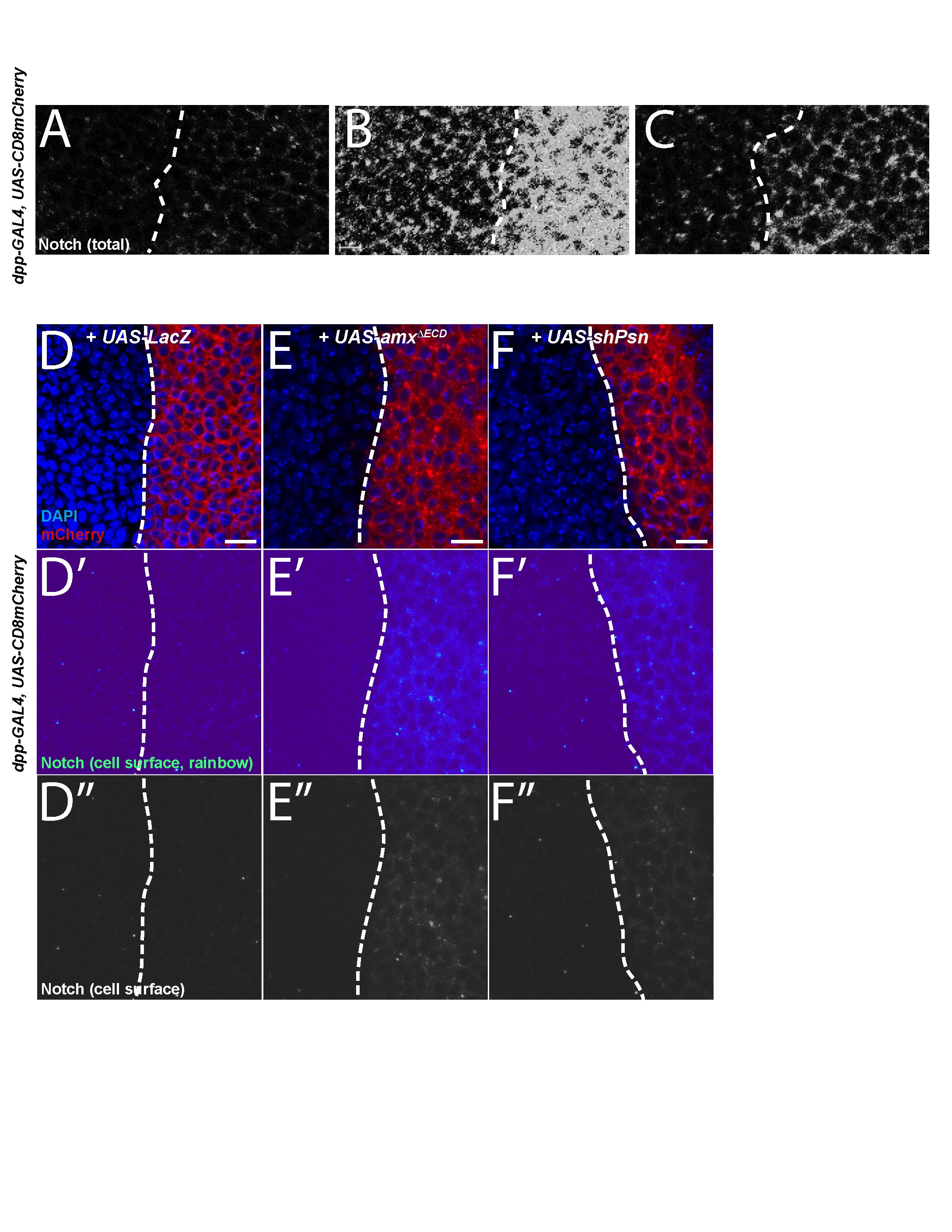

Supplement: S5 Fig — (A-C) Grey scale images of total Notch staining shown in Fig 5J’-L’, revealing incresase in Notch levels upon overexpression of AmxΔECD or Psn knockdown. (D-F”) wing disc expressing LacZ (D-D”), AmxΔECD (E-E”) or Psn-RNAi (F-F”) driven by dpp-Gal4. GAL4 expression domain is marked by mCherry (red) in D-F. Cell nuclei are marked by DAPI (blue) in D-F. Cell surface Notch is shown in rainbow in D’-F’ or grey scale in D”-F”. Notch accumulates at the cell surface upon of AmxΔECD or Psn knockdown but to a lesser extent compared to total Notch staining. Scale bars = 5 μm in (A-C). Scale bars = 10 μm in (D-F). (TIFF) [file pgen.1009962.s005.tiff]

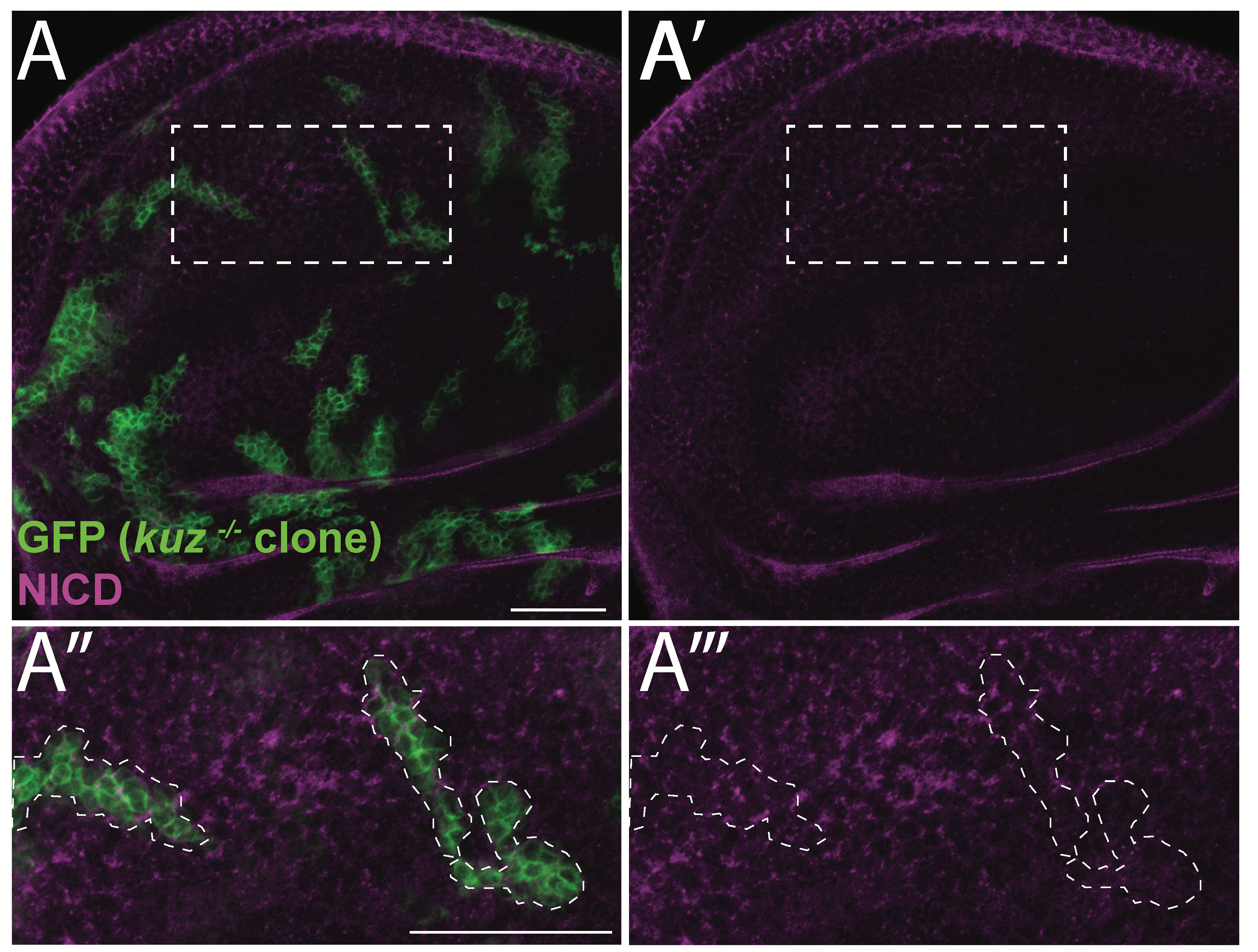

Supplement: S6 Fig — (A) kuz-/- clones (positively marked by GFP, green) were generated by MARCM using a heat shock induced Fippase (hs-FLP). The expression level and gross subcellular localization of Notch (magenta) is not altered in kuz-/- clones compared to control tissue (non-GFP cells). A” and A”‘ show the boxed region in A and A’. Scale bars = 50 μm. (TIFF) [file pgen.1009962.s006.tiff]

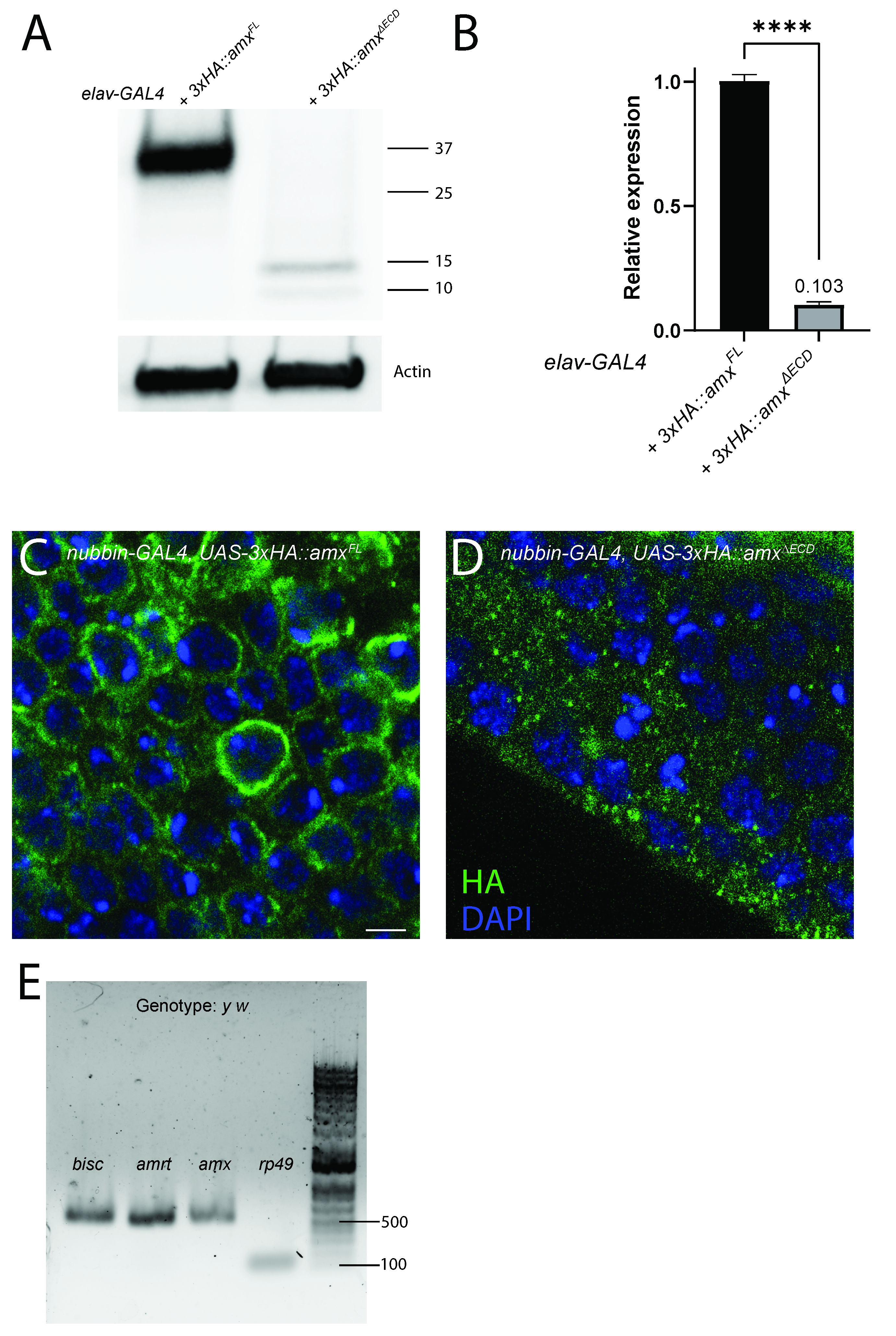

Supplement: S7 Fig — (A) Western blot of AmxFL and AmxΔECD expression driven by pan-neuronal elav-GAL4 driver. Expected band size for AmxFL is 35 kDA. Expected band size for AmxΔECD is 10 kDa. Since two bands are present around the expected size for AmxΔECD, both signals were quantified in (B). (B) AmxΔECD expression is ~10x lower than AmxFL. t-test. **** = p<0.0001. Error bars show SEM. (C-D) Immunostaining against HA tag (green) shows 3xHA::AmxFL has a membranous expression pattern (C) compared to a more punctate pattern seen with 3xHA::AmxΔECD (D). (E) RT-PCR on mRNA extracted from wing discs from control animals (genotype: y w) shows that all three TM2D genes are endogenously expressed in this tissue. Scale bars = 5 μm in (C-D). (TIFF) [file pgen.1009962.s007.tiff]

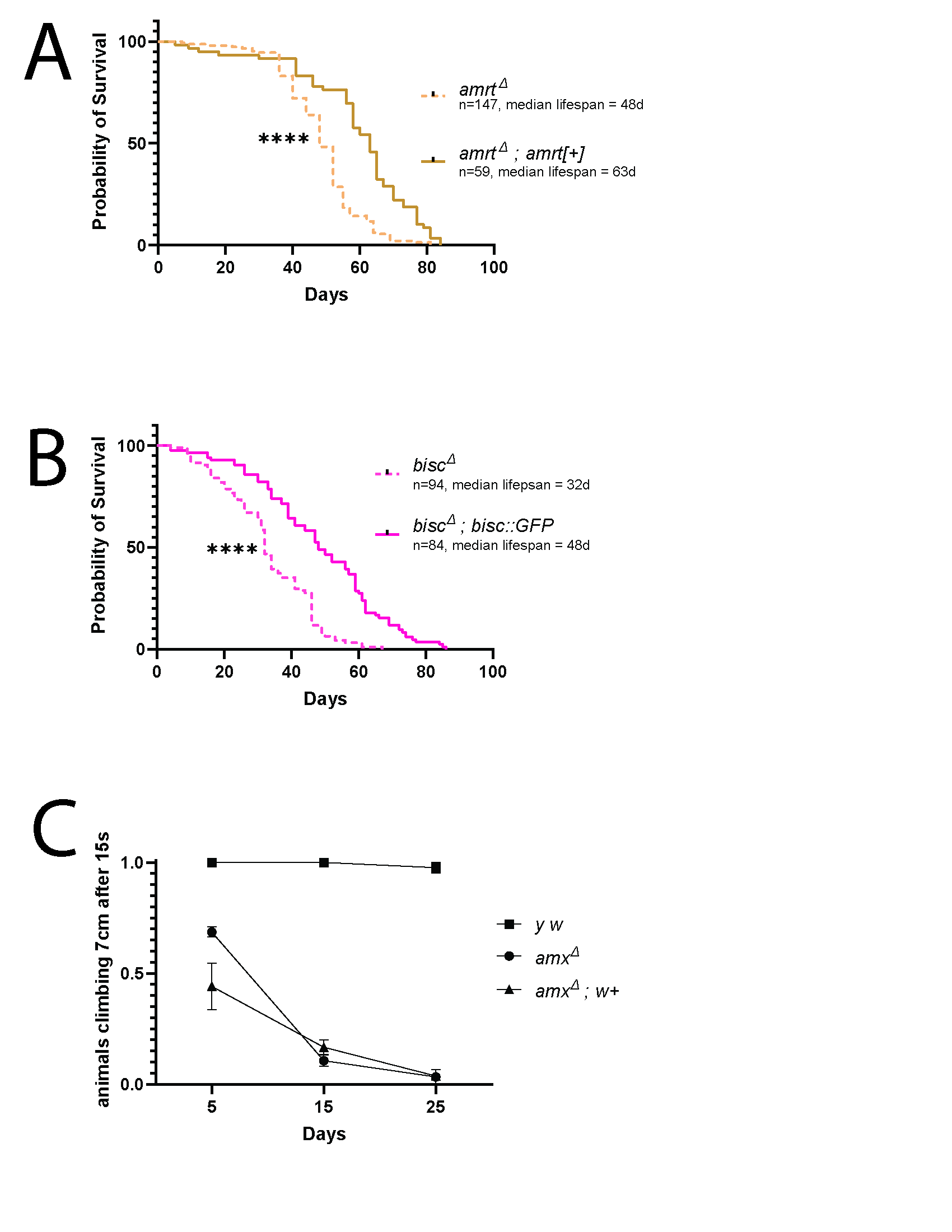

Supplement: S8 Fig — (A-B) Lifespans of amrt and bisc mutants compared to their respective controls. Animals were reared at 25°C; Log-rank test (Mantel-Cox), **** = p<0.0001. (A) amrtΔ animals (dark yellow, n = 147) have reduced lifespan compared to amrtΔ + amrt controls (pale dash yellow, n = 59). (B) biscΔ animals (dark pink, n = 94) have reduced lifespan compared to biscΔ + bisc::GFP controls (pale dash pink, n = 84). (C) amxΔ mutants (on a y w mutant background) exhibit climbing defects that worsen over time compared to controls (y w). The absence or presence of the white gene does not affect this phenotype. (TIFF) [file pgen.1009962.s008.tiff]

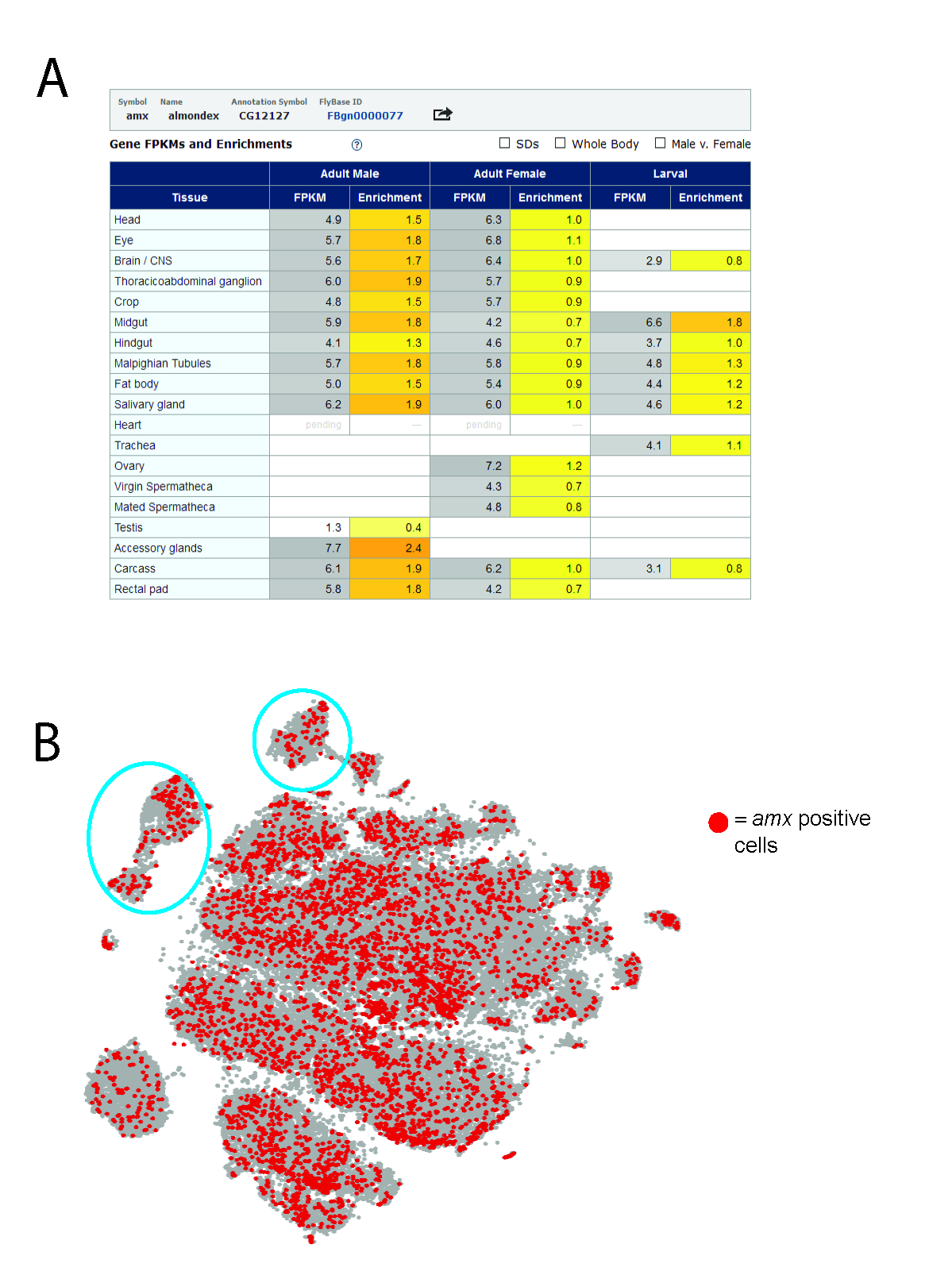

Supplement: S9 Fig — (A) Summary table for amx transcript expression provided by FlyAtlas (http://flyatlas.gla.ac.uk/FlyAtlas2/index.html?search=gene&gene=CG12127&idtype=cgnum#mobileTargetG). amx transcript is found in the Brain/CNS of adult flies, as well as other tissues. (B) Single-cell transcript data shows amx expressed in many but not all cells in the adult fly brain based on [53]. Clusters of cells positive for repo (glial marker) expression are circled in blue; the remaining cells are largely elav (neuronal marker) positive (https://scope.aertslab.org/). Red dots are cells positive for amx expression. (TIFF) [file pgen.1009962.s009.tiff]

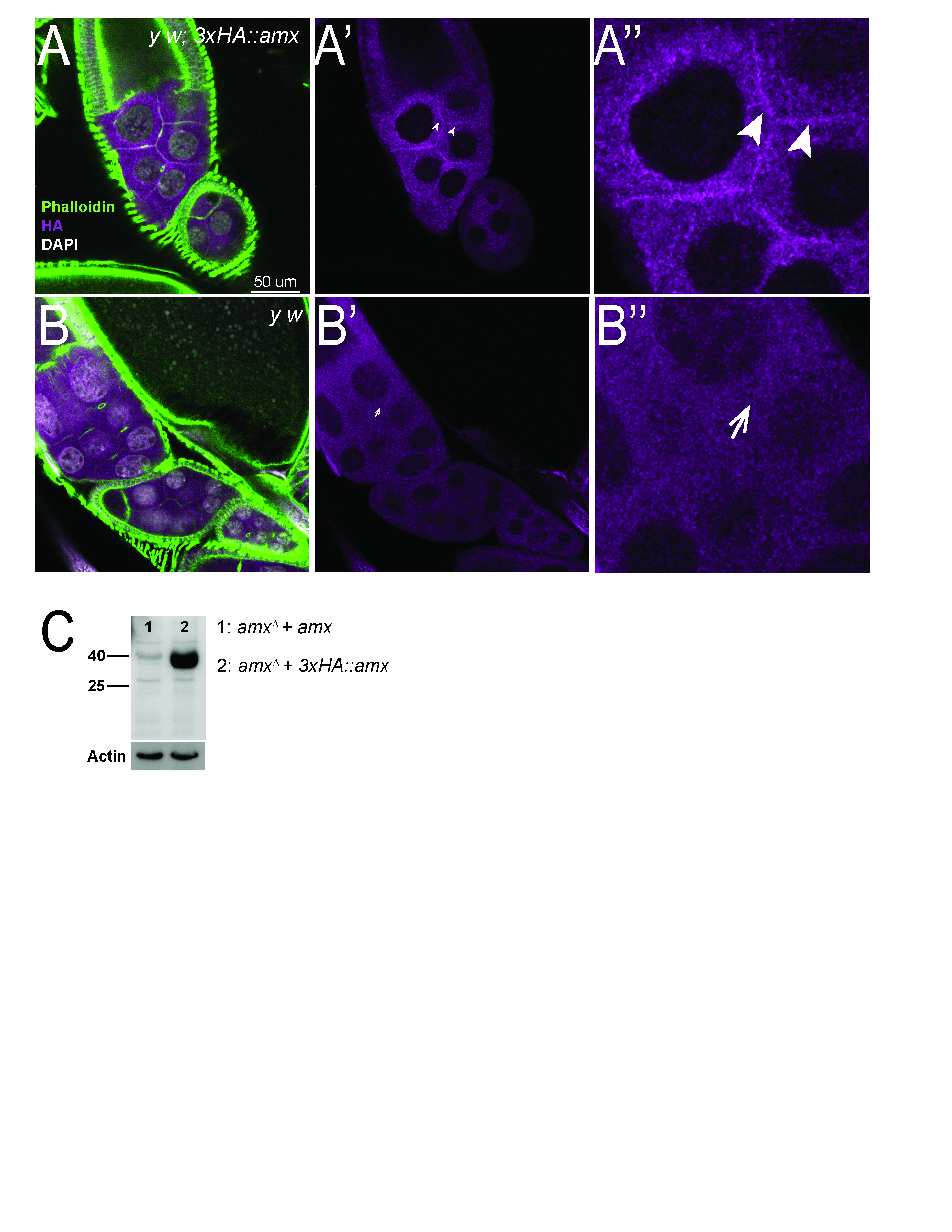

Supplement: S10 Fig — (A-B) 3xHA::Amx (magenta) localizes to the plasma membrane (marked by Phalloidin, green) separating nurse cells (arrow heads). The signal is relatively low but clearly above background levels of y w control (A” vs. B”). The same membranous localization of HA staining is not seen in negative control (arrows). (C) Western blot on ovaries showing positive expression of 3xHA::Amx (lane 2, expected size 35 kDa) in amxΔ flies compared to untagged Amx control in the same genetic background (lane 1); two ovary pairs were loaded per lane. Scale bars = 50 μm in (A-B). (TIFF) [file pgen.1009962.s010.tiff]

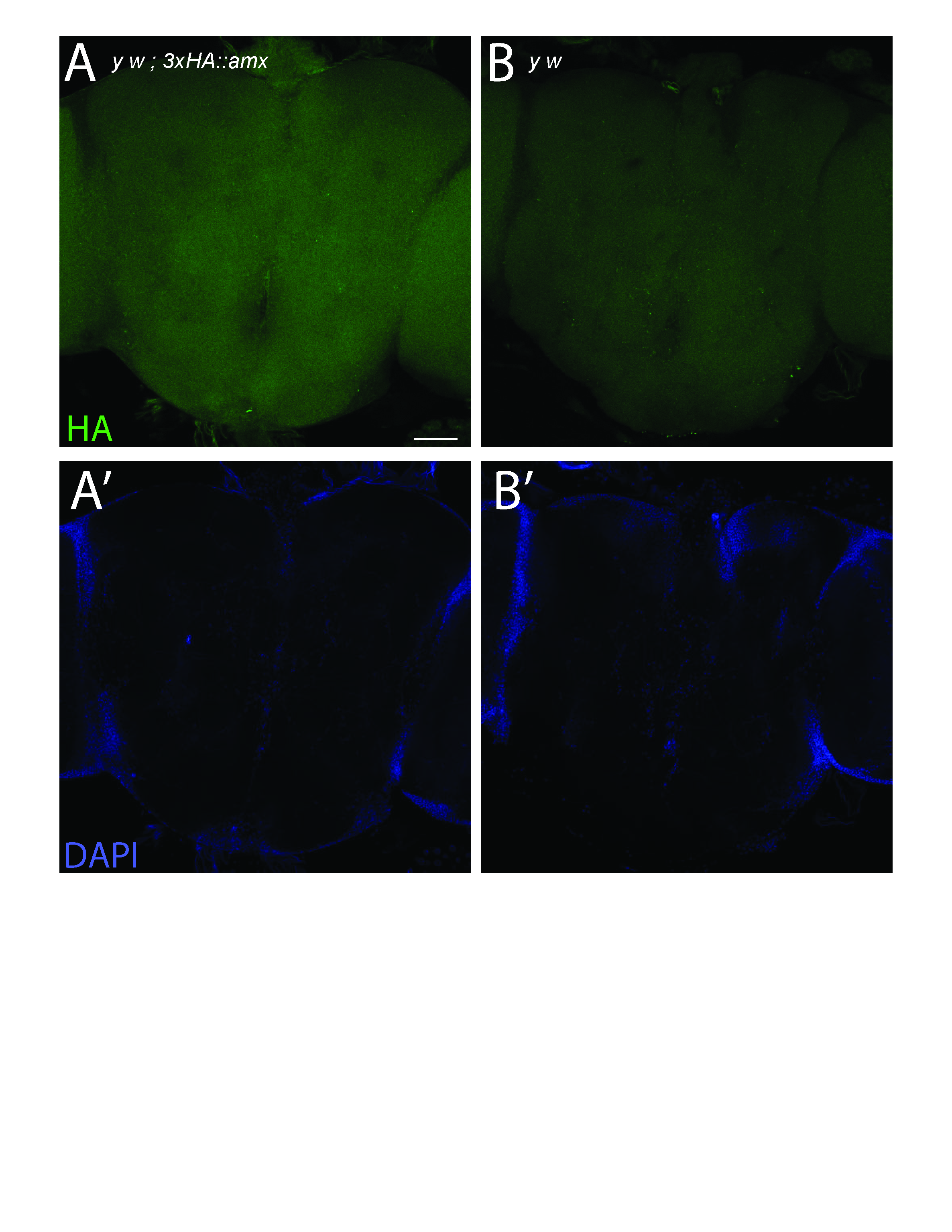

Supplement: S11 Fig — (A-B) Brains from ~1 week old animals carrying 3xHA::amxFL transgene (A) show no clear signal when immunostained for HA (green) compared to negative control (genotype: y w) brains (B). Scale bars = 50 μm in (A-B). (TIFF) [file pgen.1009962.s011.tiff]

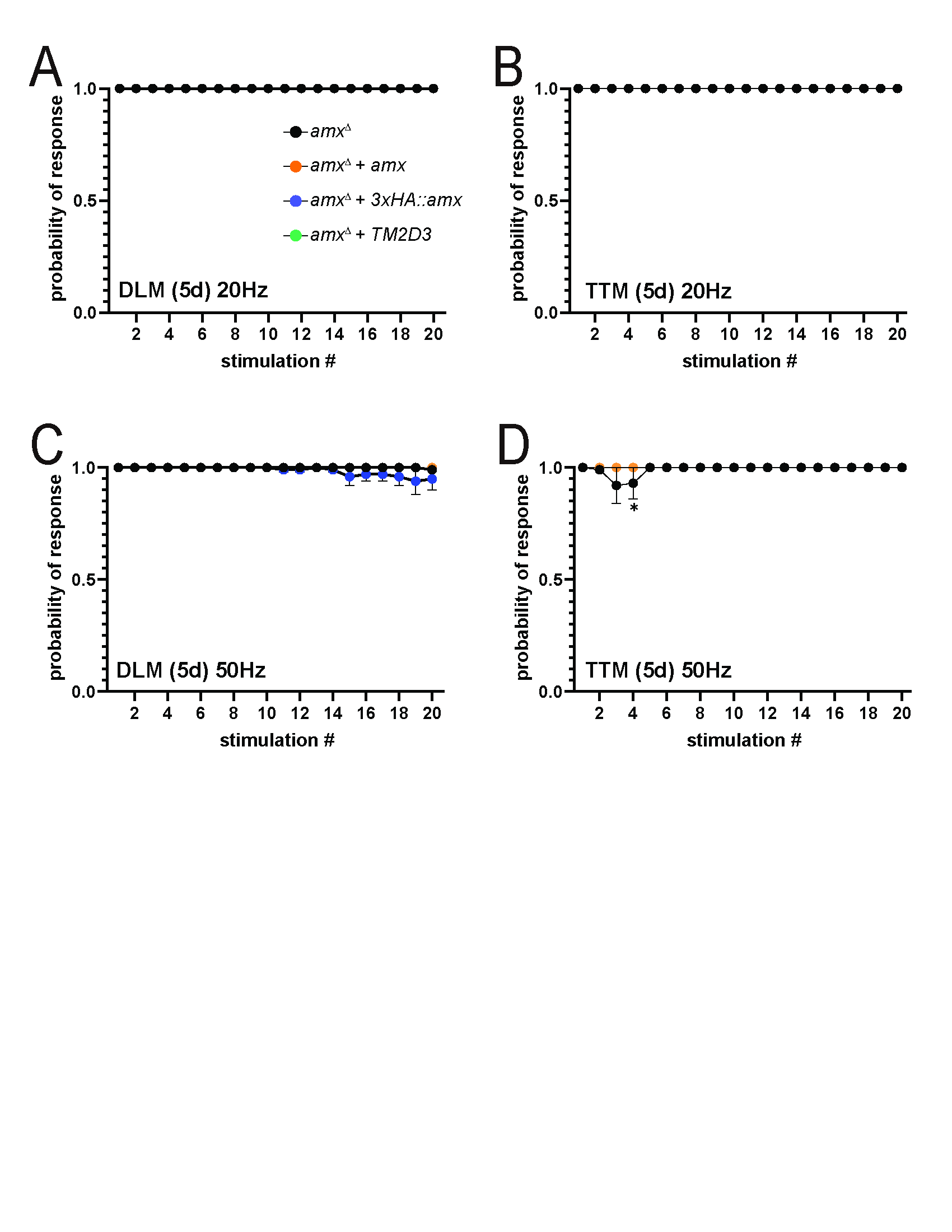

Supplement: S12 Fig — (A,C). DLM muscles of 5 day old amxΔ mutants (black) have a response similar to amxΔ + amx controls (orange) at stimulation frequencies of 20 and 50 Hz. (B,D) TTM muscles show a small but significant decrease in response probability at 50 Hz but not 20 Hz. amxΔ + 3xHA::amx (blue) flies also perform as well as controls (A-D). Multiple unpaired t-tests with Holm-Šídák correction for multiple comparisons. * = p<0.05. Error bars show SEM. (TIFF) [file pgen.1009962.s012.tiff]

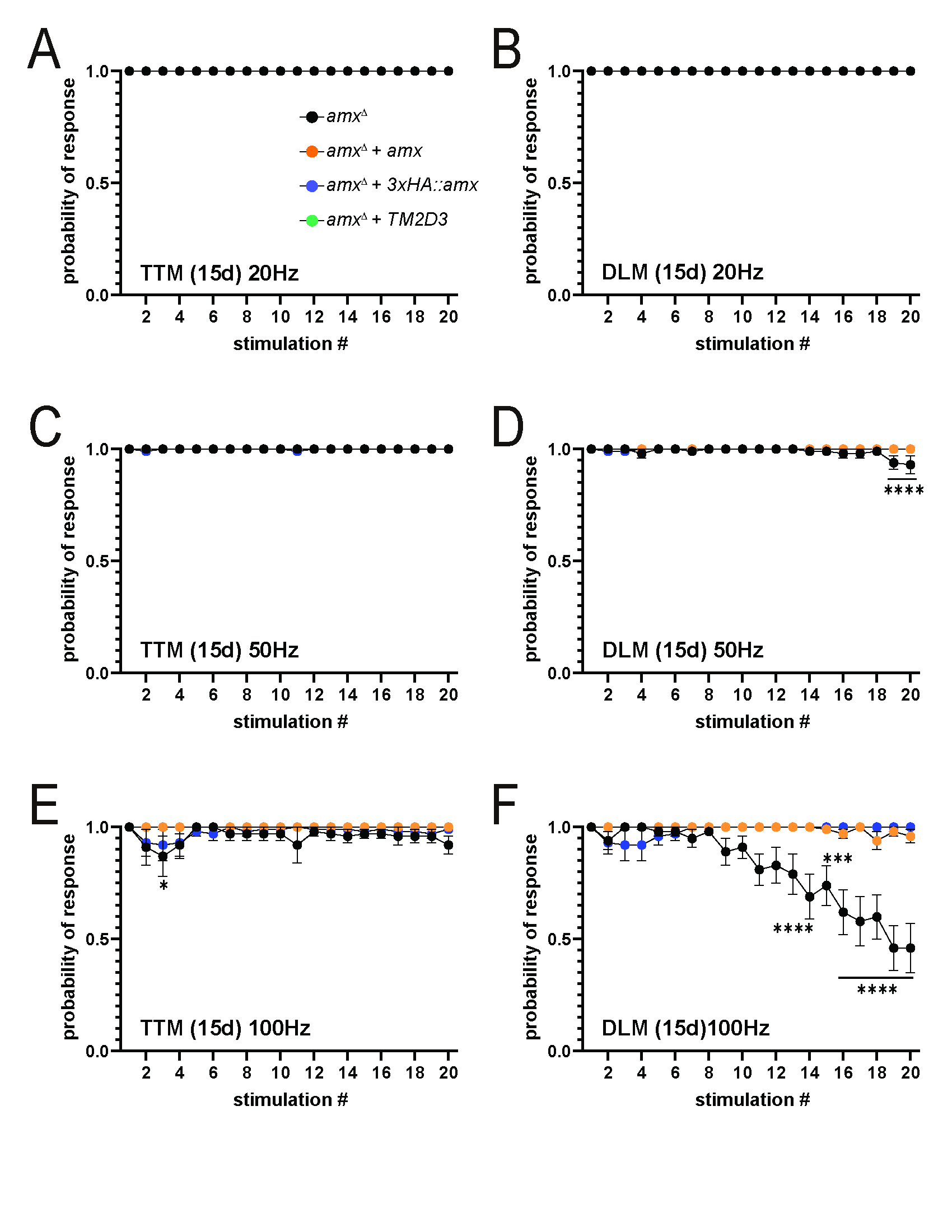

Supplement: S13 Fig — (A,C,E) TTM failure rate at 20 and 50 Hz. Responses are similar between amxΔ mutants (black) and amxΔ + amx controls (orange) (A, C), with slight but significant failures were observed at 100 Hz (E). (B,D,F) DLM failure rate at 20 and 50 Hz. amxΔ mutants have a response similar to amxΔ + amx controls at 20 Hz (B) but begin to show significant failure to respond at 50 and 100 Hz (D,F). Multiple unpaired t-tests with Holm-Šídák correction for multiple comparisons. *** = p<0.001, **** = p<0.0001. Error bars show SEM. (TIFF) [file pgen.1009962.s013.tiff]

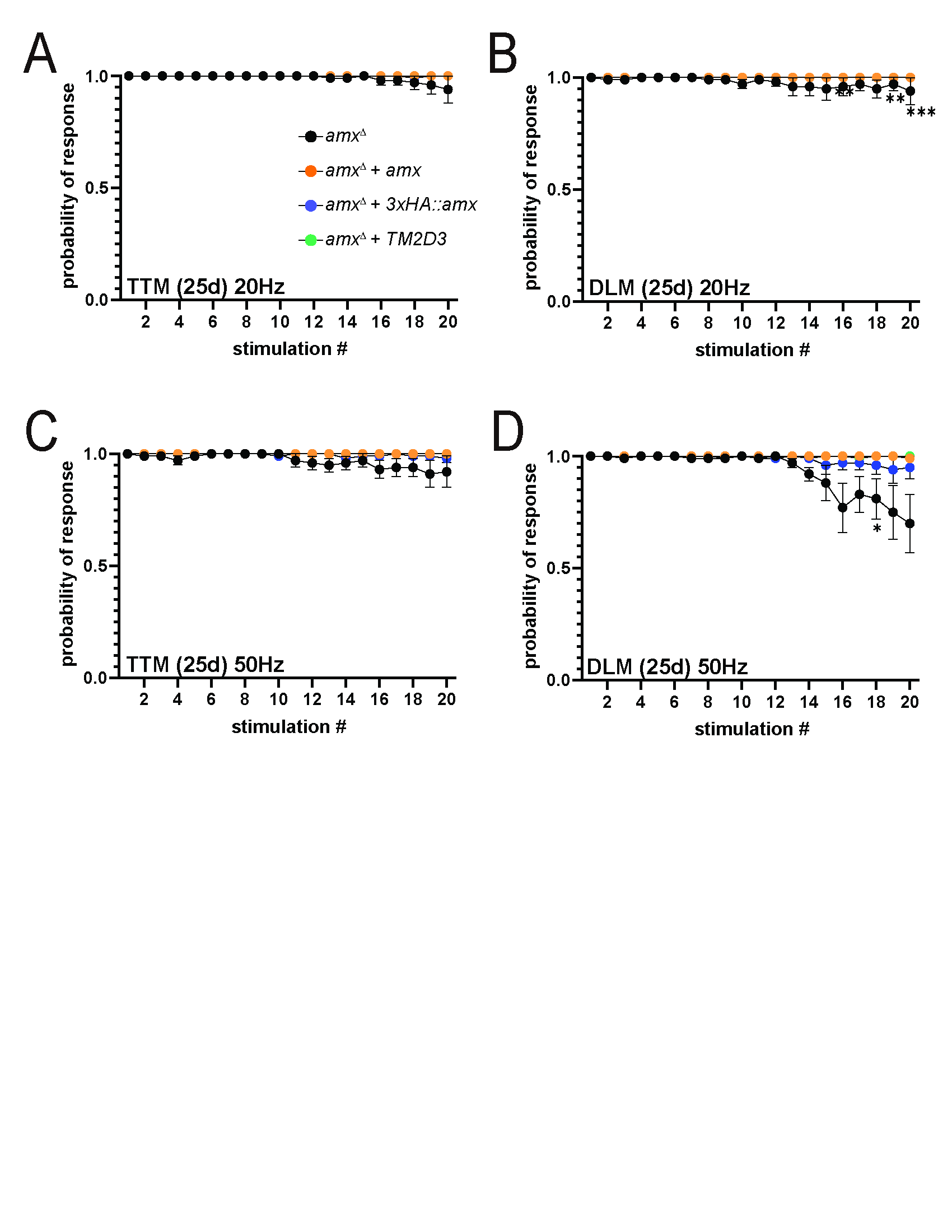

Supplement: S14 Fig — (A,C) TTM response at 20 and 50 Hz of 25 day old amxΔ mutants is similar to controls and animals carrying a human TM2D3 rescue construct. (B,D) At 25d old, amxΔ mutants perform similarly to controls at 20 Hz (B) but show a significant increase in response failure at 50 Hz (D). Human TM2D3 rescued flies again perform similarly to controls. Multiple unpaired t-tests with Holm-Šídák correction for multiple comparisons. * = p<0.05. ** = p<0.01, *** = p<0.001. Error bars show SEM. (TIFF) [file pgen.1009962.s014.tiff]
