# Supplementary material for: TM2D genes regulate Notch signaling and neuronal function in Drosophila
Source: PLoS Genet. 2021 Dec 14;17(12):e1009962. doi: 10.1371/journal.pgen.1009962 (PMC8714088; doi:10.1371/journal.pgen.1009962)
Supplement: S1 Table — The following abbreviations are used. PMID: PubMed ID (https://pubmed.ncbi.nlm.nih.gov), BDSC: Bloomington Drosophila Stock Center ID (https://bdsc.indiana.edu), DSHB: Developmental Studies Hybridoma Bank ID (https://dshb.biology.uiowa.edu) (DOCX) [file pgen.1009962.s015.docx]

**Supplemental Table 1. Key resources used in this study.** The following abbreviations are used. PMID: PubMed ID ([https://pubmed.ncbi.nlm.nih.gov](https://pubmed.ncbi.nlm.nih.gov/)), BDSC: Bloomington Drosophila Stock Center ID ([https://bdsc.indiana.edu](https://bdsc.indiana.edu/)), DSHB: Developmental Studies Hybridoma Bank ID ([https://dshb.biology.uiowa.edu](https://dshb.biology.uiowa.edu/))

| **Reagent type (*species*) or resource** | **Designation** | **Source or reference** | **Identifiers (FlyBase)** | **Additional information** |
| --- | --- | --- | --- | --- |
| Genetic reagent (*D. melanogaster*) | *amx^Δ^*  *(a.k.a. amx^ΔCDS^)* | PMID: 30091705 | FBal0341603 | knockout of amx using the *y^wing2+^* marker |
|  | *amrt^Δ^* | This study |  | knockout of amrt using the *y^body+^* marker |
|  | *bisc^Δ^* | This study |  | knockout of bisc using the *y^body+^* marker |
|  | *pattB-amx*  (a.k.a. amx^+t3.325^) | PMID: 27764101 | FBal0338083 | Untagged genomic rescue transgene for *amx*, injected into VK37 |
|  | *pattB-3xHA::amx* | This study |  | 3xHA-tagged genomic rescue transgene for *amx*, injected into VK37 |
|  | *pattB-TM2D3*  (a.k.a. *TM2D3^amx.1^*) | PMID: 27764101 | FBal0338084 | Humanized genomic rescue transgene for *amx* |
|  | *pattB-amrt* | This study |  | Untagged genomic rescue transgene for *amrt*, injected into VK37 |
|  | *pattB[w+]* | This study |  | Empty pattB plasmid injected into VK37 docking site |
|  | *bisc::GFP* | This study |  | GFP(and other epitobe)-tagged genomic rescue transgene for *bisc*. Derived from fosmid: FlyFos021003 (PMID: 26896675) , injected into VK33 |
|  | *y, w, iso#6(X); ;attP2{nos-Cas9}* | PMID: 30091705 |  | CRISPR stock used to generate *amx^Δ^* and *amrt^Δ^*. Derived from RRID:BDSC_78782 (X-chromosome isogenized) |
|  | *y, w; iso#2(2); attP2{nos-Cas9}* | PMID: 30091705 |  | CRISPR stock used to generate *bisc^Δ^*. Derived from RRID:BDSC_78782 (2^nd^ chromosome isogenized) |
|  | *y, M{vas-int.Dm}ZH-2A, w; PBac{y[+]-attP-3B}VK00037* | PMID: 17138868, BDSC: 24872 | FBti0099694, FBti0076455 | phiC31 stock used to generate transgenes on the 2^nd^ chromosome (a.k.a. VK37). |
|  | *y, M{vas-int.Dm}ZH-2A, w; PBac{y[+]-attP-3B}VK00033* | PMID: 17138868, BDSC: 24871 | FBti0099694, FBti0076453 | phiC31 stock used to generate transgenes on the 3^rd^ chromosome (a.k.a. VK33). |
|  | *y, w; UAS-amx* | This study |  | UAS transgene expressing full length untagged Amx, inserted into VK37 |
|  | *y, w; UAS-3xHA::amx^FL^* | This study |  | UAS transgene expressing full length 3xHA-tagged Amx, inserted into VK37 |
|  | *y, w; UAS-3xHA::amx*^Δ^*^ECD^* | This study |  | UAS transgene expressing truncated 3xHA-tagged Amx, inserted into VK37 |
|  | *y, w; UAS-N^FL^* | This study |  | UAS transgene expressing full length Notch, inserted into VK37 |
|  | *y, w; UAS-N^ΔEGF1-18.LNR^* | This study |  | UAS transgene expressing Notch that depends on S2 and S3 cleavages, inserted into VK37 |
|  | *y, w; UAS-N^EXT^* | This study |  | UAS transgene expressing Notch that depends on S3 celavage, inserted into VK37 |
|  | *y, w; UAS-N^ICD^* | This study |  | UAS transgene expressing intracellular domain of Notch, inserted into VK37 |
|  | *UAS-CD8::mCherry(2) (a.k.a. w; P{w[+mC]=UAS-mCD8.ChRFP}2)* | BDSC: 27391 | FBst0027391 | UAS transgene expressing membrane tethered mCherry |
|  | *UAS-CD8::mCherry(3) (a.k.a. w; P{w[+mC]=UAS-mCD8.ChRFP}3)* | BDSC: 27392 | FBst0027392 | UAS transgene expressing membrane tethered mCherry |
|  | *UAS-shPsn (a.k.a. UAS-Psn.shRNA.3)* | PMID: 28495961 | FBal0327448 | UAS transgene expressing shRNA agaist *Psn*. Gift from Drs. Jongkyun Kang and Jie Shen |
|  | *UAS-LacZ* | PMID: 8223268 | FBal0042106 | UAS transgene expressing LacZ (negative control). Gift from Dr. Hugo Bellen |
|  | *w; P{w[+mW.hs]=en2.4-GAL4}e16E, P{w[+mC]=UAS-myr-mRFP}1, P{w[+m*]=NRE-EGFP.S}5A* | PMID: 22384384, BDSC: 30729 | FBti0003572, FBti0027895, FBti0130022 | *en-GAL4* line with *UAS-RFP* and *NRE-GFP* |
|  | *w; P{w[nub.PK]=nub-GAL4.K}2* | BDSC: 86108 | FBti0150342 | *nub-GAL4* line |
|  | *y, w; P{w[+mW.hs]=GawB}pnr[MD237]/TM3, P{w[+mC]=UAS-y.C}MC2, Ser[1]* | BDSC: 3039 | FBti0004011 | *pnr-GAL4* line |
|  | *hsFLP; tub-Gal80[ts], FRT40A/CyO ; tub-Gal4, UAS-GFP/TM6b Tb* | PMID: 29773559 |  | MARCM line. Gift from Dr. Wu-Min Deng |
|  | *kuz^e29-4^, FRT40A/CyO* | This study | FBal0051471, FBti0002071 | *kuz^e29-4^* (from RRID: BDSC_5804) was recombined onto *FRT40A* |
|  | *P{GawB}elav^C155^* | BDSC: 458 | FBti0002575 | *elav-GAL4* (a.k.a. C155) line |
|  | *P{nSyb-GAL4.S}3* | BDSC: 51635 | FBti0150361 | *nSyb-GAL4* line |
| Primary Antibody | mouse anti-Notch intracellular domain (clone C17.9C6) | DSHB: C17.9C6 |  | monoclonal antibody against the intracellular domain of *Drosophila* Notch |
|  | mouse anti-Notch extracellular domain (clone C458.2H) | DSHB: C458.2H |  | monoclonal antibody against the extracellular domain of *Drosophila* Notch (EGF repeats 12-20) |
|  | mouse anti-Cut (clone 2B10) | DSHB: 2B10 |  | monoclonal antibody against *Drosophila* Cut |
|  | rat anti-HA (clone 3F10) | Sigma-Aldrich: 11867423001 |  | monoclonal antibody against the HA peptide |
| Secondary Antibody | donkey anti-rat IgG-Cy3 | Jackson ImmunoResearch: 712-165-153 |  | secondary antibody for immunostaining |
|  | donkey anti-mouse IgG-Alexa-647 | Jackson ImmunoResearch: 715-605-151 |  | secondary antibody for immunostaining |
|  | donkey anti-rat HRP | Jackson ImmunoResearch: 712-035-150 |  | secondary antibody for Western blot |
