# Supplementary material for: TM2D genes regulate Notch signaling and neuronal function in Drosophila
Source: PLoS Genet. 2021 Dec 14;17(12):e1009962. doi: 10.1371/journal.pgen.1009962 (PMC8714088; doi:10.1371/journal.pgen.1009962)
Supplement: S2 Table — (DOCX) [file pgen.1009962.s016.docx]

**Supplemental Table 2. Genotypes of the flies shown in each figure panel.**

| **Figure & Panel #** | **Genotype** |
| --- | --- |
| **Figure 1** | |
| **C,D** | Embryos from *y, w, amx^Δ^* |
| **C,E** | Embryos from *y, w, amx^Δ^ ; amx* |
| **C,F** | Embryos from *y, w, amx^Δ^ ; 3xHA::amx* |
| **C,G** | Embryos from *y, w, amx^Δ^ ; TM2D3* |
| **Figure 2** | |
| **C,E** | Embryos from *y, w, amrt^Δ^* |
| **D,E** | Embryos from *y, w, amrt^Δ^ ; amrt* |
| **G,I** | Embryos from *y, w; bisc^Δ^* |
| **H,I** | Embryos from *y, w; bisc^Δ^ ; bisc::GFP* |
| **Figure 3** | |
| **B** | Embryos from *y, w* |
| **C** | Embryos from *y, w, amx^Δ^, amrt^Δ^* |
| **D** | Embryos from *y, w, amx^Δ^, amrt^Δ^ ; bisc^Δ^* |
| **E,H,K** | *y, w* |
| **F,I,L** | *y, w, amx^Δ^, amrt^Δ^* |
| **G,J,M** | *y, w, amx^Δ^, amrt^Δ^ ; bisc^Δ^* |
| **Figure 4** | |
| **B** | y*, w; UAS-3xHA::amx^FL^ / +; pnr-GAL4/+* |
| **C,G** | *en-GAL4, UAS-myr::mRFP, NRE-GFP / UAS-3xHA::amx^FL^* |
| **E** | *y, w; UAS-3xHA::amx^ΔECD^ / +; pnr-GAL4/+* |
| **F,H** | *en-GAL4, UAS-myr::mRFP, NRE-GFP / UAS-3xHA::amx^ΔECD^* |
| **Figure 5** | |
| **B** | *nub-GAL4, UAS-CD8::mCherry / UAS-LacZ* |
| **C,I** | *nub-GAL4, UAS-CD8::mCherry / UAS-N^ICD^* |
| **D,I** | *nub-GAL4, UAS-CD8::mCherry / UAS-N^EXT^* |
| **E,I** | *nub-GAL4, UAS-CD8::mCherry / UAS-N^ΔEGF1-18.LNR^* |
| **F,I** | *nub-GAL4, UAS-3xHA::amx^ΔECD^ / UAS-N^ICD^* |
| **G,I** | *nub-GAL4, UAS-3xHA::amx^ΔECD^ / UAS-N^EXT^* |
| **H,I** | *nub-GAL4, UAS-3xHA::amx^ΔECD^ / UAS-N^ΔEGF1-18.LNR^* |
| **J** | *dpp-GAL4, UAS-CD8::mCherry / UAS-lacZ* |
| **K** | *UAS-3xHA::amx^ΔECD^/+; dpp-GAL4, UAS-CD8::mCherry/+* |
| **L** | *dpp-GAL4, UAS-CD8::mCherry / UAS-shPsn* |
| **Figure 6** | |
| **A,C,E,F,G,H** | *y, w, amx^Δ^* |
| **A,E,F,G,H** | *y, w, amx^Δ^ ; amx* |
| **A,E,F,G,H** | *y, w, amx^Δ^ ; TM2D3* |
| **B** | *y, w, amx^Δ^ ; UAS-LacZ ; nSyb-GAL4* |
| **B** | *y, w, amx^Δ^ ; UAS-amx ; nSyb-GAL4* |
| **C,E,F,G,H** | *y, w, amx^Δ^ ; 3xHA::amx* |
| **Sup. Figure 2** | |
| **A, B** | *y, w* |
| **A** | *y, w, amx^Δ^* |
| **A** | *y, w, amrt^Δ^* |
| **A** | *y, w; bisc^Δ^* |
| **B** | *y, w, amx^Δ^, amrt^Δ^* |
| **B** | *y, w, amx^Δ^, amrt^Δ^ ; bisc^Δ^* |
| **Sup. Figure 3** | |
| **A** | *nub-GAL4, UAS-CD8::mCherry / UAS-N^FL^* |
| **B** | *nub-GAL4, UAS-3xHA::amx^ΔECD^ / UAS-N^FL^* |
| **C,E** | *nub-GAL4, UAS-CD8::mCherry / UAS-LacZ* |
| **D,E** | *nub-GAL4, UAS-CD8::mCherry / UAS-N^ICD^* |
| **Sup. Figure 4** | |
| **A** | *nub-GAL4 / UAS-3xHA::amx^ΔECD^* |
| **B** | *nub-GAL4, UAS-3xHA::amx^ΔECD^* / *nub-GAL4, UAS-3xHA::amx^ΔECD^* |
| **C** | *UAS-3xHA::amx^FL^* /+ ; *dpp-GAL4/+* |
| **D** | *UAS-3xHA::amx^ΔECD^/+* ; *dpp-GAL4/+* |
| **Sup. Figure 5** | |
| **A,D** | *dpp-GAL4, UAS-CD8::mCherry / UAS-lacZ* |
| **B,E** | *UAS-3xHA::amx^ΔECD^/+; dpp-GAL4, UAS-CD8::mCherry/+* |
| **C,F** | *dpp-GAL4, UAS-CD8::mCherry / UAS-shPsn* |
| **Sup. Figure 6** | |
| **A** | *hsFLP; tub-Gal80^ts^, FRT40A / kuz^e29-4^, FRT40A; tub-GAL4, UAS-GFP/+* |
| **Sup. Figure 7** | |
| **A,B** | *elav-GAL4 ; UAS-3xHA::amx^FL^* |
| **A,B** | *elav-GAL4 ; UAS-3xHA::amx^ΔECD^* |
| **C,D** | *nub-GAL4 / UAS-3xHA::amx^FL^* |
| **C,D** | *nub-GAL4 / UAS-3xHA::amx^ΔECD^* |
| **E** | *y, w* |
| **Sup. Figure 8** | |
| **A** | *y, w, amrt^Δ^* |
| **A** | *y, w, amrt^Δ^ ; amrt* |
| **B** | *y, w; bisc^Δ^* |
| **B** | *y, w; bisc^Δ^ ; bisc::GFP* |
| **C** | *y, w* |
| **C** | *y, w, amx^Δ^* |
| **C** | *y, w, amx^Δ^ ; pattB[w^+^]* |
| **Sup. Figure 10** | |
| **A** | *y, w, amx^Δ^ ; 3xHA::amx* |
| **B** | *y, w* |
| **C** | *y, w, amx^Δ^ ; amx* |
| **C** | *y, w, amx^Δ^ ; 3xHA::amx* |
| **Sup. Figure 11** | |
| **A** | *y, w, amx^Δ^ ; 3xHA::amx* |
| **B** | *y, w* |
| **Sup. Figure 12** | |
| **A,B,C,D** | *y, w, amx^Δ^* |
| **A,B,C,D** | *y, w, amx^Δ^ ; amx* |
| **A,B,C,D** | *y, w, amx^Δ^ ; 3xHA::amx* |
| **A,B,C,D** | *y, w, amx^Δ^ ; TM2D3* |
| **Sup. Figure 13** | |
| **A,B,C,D,E,F** | *y, w, amx^Δ^* |
| **A,B,C,D,E,F** | *y, w, amx^Δ^ ; amx* |
| **A,B,C,D,E,F** | *y, w, amx^Δ^ ; 3xHA::amx* |
| **A,B,C,D,E,F** | *y, w, amx^Δ^ ; TM2D3* |
| **Sup. Figure 14** | |
| **A,B,C,D** | *y, w, amx^Δ^* |
| **A,B,C,D** | *y, w, amx^Δ^ ; amx* |
| **A,B,C,D** | *y, w, amx^Δ^ ; 3xHA::amx* |
| **A,B,C,D** | *y, w, amx^Δ^ ; TM2D3* |
